# Cancer, cardiovascular disease, and all-cause mortality in Iraqi- and Swedish-born individuals in Sweden: the MEDIM cohort study

**DOI:** 10.1038/s41598-023-33379-6

**Published:** 2023-04-15

**Authors:** Nadine Fadhel Dhaher, Miriam Pikkemaat, Nael Shaat, Anton Nilsson, Louise Bennet

**Affiliations:** 1 Genomics, Diabetes and Endocrinology, Department of Clinical Sciences, Lund University, Malmö, Sweden; 2 Department of Endocrinology, Skåne University Hospital, Jan Waldenströms gata 24, 205 02 Malmö, Sweden; 3 Department of Clinical Sciences Malmö, Centre for Primary Health Care Research, Lund University, Malmö, Sweden; 4 Department of Laboratory Medicine, Lund University, Lund, Sweden; 5 Department of Clinical Sciences in Malmö, Lund University, Malmö, Sweden; 6 Clinical Research and Trial Centre, Lund University Hospital, Lund, Sweden

**Keywords:** Cancer epidemiology, Endocrine system and metabolic diseases

## Abstract

Immigrants from the Middle East to Sweden have a twice as high prevalence of type 2 diabetes (T2D) and obesity as native-born Swedes. Both obesity and T2D have been linked to increased incidence of cancer, cardiovascular disease (CVD) and all-cause mortality (ACM); however, data on differences between ethnicities are scarce. In a population-based cohort we aimed to study the impact of Middle Eastern and European ethnicity on ACM, cancer- and CVD related mortality, incidence of cancer and CVD in an eight-year follow-up study. Methods: People born in Iraq or Sweden, who were 30–75 years of age, were invited from 2010 to 2012 to participate in the population based MEDIM study including a health exam, fasting blood sampling, assessment of insulin secretion and action (through oral glucose tolerance test) and questionnaires assessing history of CVD, cancer and T2D. Register data were retrieved from baseline until the 31st of December 2018 from the Swedish National Patient Register and Cause of Death register regarding CVD diagnosis, cancer diagnosis and cause of death. Information regarding diabetes diagnosis was retrieved from the National Diabetes Register. Individuals with a history of cancer or CVD at baseline were excluded. Cox regression analysis was assessed to study the adjusted hazard ratios (HR) for the relationships between ethnicity and ACM, cancer events, CVD events, death from cancer, and death from CVD, with adjustments for age, sex, anthropometrical measures, T2D and lifestyle. A total of 1398 Iraqi- and 757 Swedish-born residents participated in the study. ACM was considerably lower in Iraqi- compared to Swedish-born individuals HR 0.32 (95% CI 0.13–0.79) (p < 0.05). Furthermore, cancer related morbidity and mortality HR 0.39 (0.22–0.69) (p < 0.01) as well as CVD related morbidity and mortality HR 0.56 (0.33–0.95) (p < 0.05) were lower in the Iraqi-born group compared to the Swedish-born group for. The differences in mortality and cancer rates across ethnicities are not fully explained by anthropometric, environmental or metabolic measures but lie elsewhere. Further studies are needed to increase the understanding of contributing mechanisms.

## Introduction

According to data derived from Statistics Sweden (2020), a total of 19.6% of the Swedish population consists of individuals born outside Sweden^1^. Immigration and urbanisation are two well-known risk factors for developing obesity and T2D^2,3^ and immigrants from the Middle East to Sweden are more insulin resistant and have a twice as high prevalence of T2D as well as obesity compared to native Swedes^3–5^. Affluence and western lifestyles, which involve excess energy intake, are some of the reasons behind the higher rates of cancer in Sweden and many developed countries^6,7^.

The relationship between high BMI, T2D and cancer is thought to be mediated by several mechanisms including hyperinsulinemia and dyslipidaemia^8^.

Since T2D is a strong risk factor for CVD and excess body weight has been associated with an increased risk of cancer incidence and mortality^9–11^, it is rather surprising that in Swedish nationwide data, non-Western immigrants with T2D displayed a 30–60% lower risk of ACM, and cause specific mortality (CSM) in cerebrovascular, diabetes-related and death from cancer^12–14^.

There are only a few studies that have addressed the incidence of cancer among immigrants from the Middle East region compared to that of the endogenous population, and these have shown diverse results. For instance, Arabic females tend to have a higher risk of thyroid cancer while the incidence rates of other cancer forms vary a lot but tend to be similar to other migrant groups and, in some cases, even similar incidence rates to the endogenous population^15,16^. As for immigrants to Sweden, cancer incidence has been shown to vary greatly with the lowest cancer standardised incidence ratio found among Iraqi men for 18 cancer sites^17^. Apart from the Swedish studies, several international studies have suggested a lower incidence of cancer among Middle Eastern immigrants to Europe, Australia, and Canada^18–22^. Studies on immigrant minorities have also revealed ethnic differences in the incidence of CVD with better baseline CV health and lower incidence of CVD compared to the host population in some studies while others have reported elevated risks of CVD^23–25^. The incidence of CVD among immigrants has also been linked to the length of stay in the host country with a higher incidence of CVD among immigrants with longer residence, which indicates cultural orientation towards the host culture^23,26^.

The high prevalence of T2D and obesity amongst the Middle Eastern immigrant groups raises the question if ACM, the incidence of cancer and CVD in this growing population of immigrants from the Middle East differs compared to the native-born population.

### Aims

Based on the MEDIM cohort we aimed to study if Middle Eastern ethnicity influences ACM, CSM and the incidence of cancer- and CVD morbidity respectively with adjustment for anthropometrical measures, and lifestyle behaviour.

## Material and methods

### The MEDIM Study Cohort

**Figure 1:**
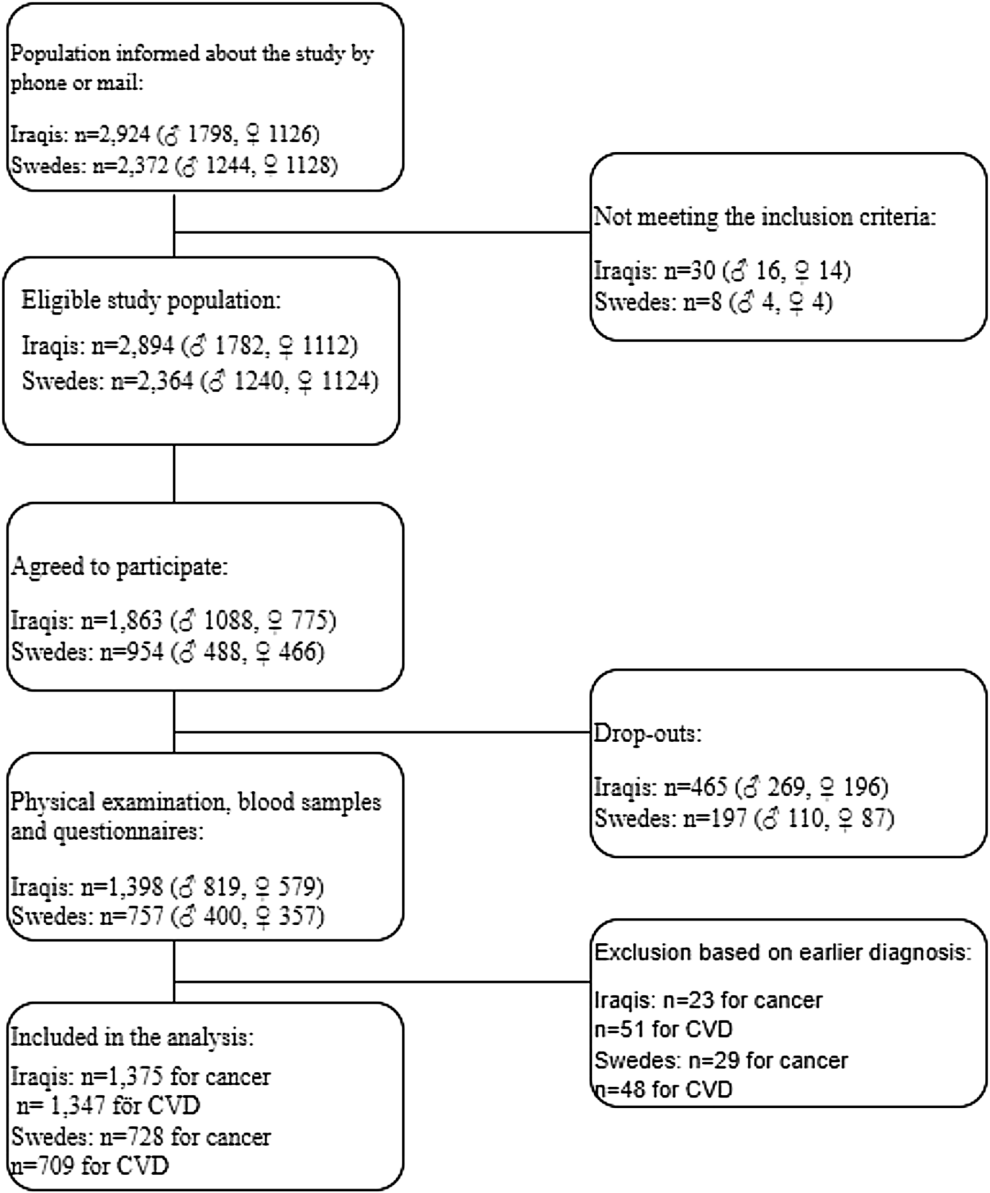
A flowchart describing the recruitment of MEDIM participants, response rate and participants included in the analysis.

The baseline investigations in the MEDIM cohort were conducted between 2010 and 2012 in the city of Malmö, Sweden. The study cohort has been described in more detail in previous publications^4^. Briefly, people born in Iraq or Sweden 30–75 years of age were selected randomly from the Swedish Population Register and invited by phone and mail to approve their participation. The individuals were also matched for sex and 10-year age distributions (2:1 matching)^4^. A total of 2,924 Iraqis and 2,372 Swedes were informed about the study, 38 residents (Iraq n = 30, Sweden n = 8) did not meet the inclusion criteria and due to dropouts (people not attending the health exam), hence the study population was reduced by 465 Iraqis and 197 Swedes. A flowchart describing the recruitment of MEDIM participants and response rate as well as the participants included in the analysis is provided in Fig. 1.

People with type 1 DM, severe physical or mental illness or disabilities were not included in the study. The remaining 1398 Iraqi- and 757 Swedish-born individuals represent the population of the current study.

Participants with no history of diabetes diagnosis underwent oral glucose tolerance tests (OGTT) and blood samples were collected prior to glucose load at 30, 60, 90 and 120 min.

The physical examinations and blood samples were conducted and collected by trained Swedish- and Arabic-speaking research nurses. Enzymatic methods were used to estimate plasma HDL-cholesterol (Boehringer Mannheim GmbH, Germany) and triglyceride levels (Bayer Diagnostics)^27^ whereas Friedewald's equation was used to estimate plasma LDL-cholesterol levels^28^.

Questionnaires were filled out by the participants providing information on lifestyle habits, family history of DM, previous diagnosis of T2D, cancer, previous diagnosis of CVD as well as present medication and family history of DM^5^. Participants were asked how many standard glasses of alcohol they usually consumed during an average week. One standard glass was equal to 50 centilitres (cL) of beer (3.5% alcohol by volume (abv)), 25 cL of strong beer (≥ 4.5% abv), 12–15 cL of wine (12–15% abv), 8 cL of wine (≥ 16 abv), or 4 cL of liquor^29^. Those consuming any amount of alcohol were considered alcohol consumers.

Follow-up: The time of observation of the study individuals was from the day of enrolment in the MEDIM baseline study until the 31st of December 2018. Information on CV events, cancer diagnoses and cause of death were retrieved from the National Patient Register (NPR) and the Cause of Death Register (CDR).

In this study, the ICD 10 (International Statistical Classification of Diseases and Related Health Problems 10th revision) was used with codes from *chapter C* for cancer disease and death from cancer and *chapter I* (Disease of the circulatory system) for CVD and death from CVD.

ICD 10 codes I210, I211, I214, I213, I219, I220 and I252 defined myocardial infarction and I259 was additionally included when defining death from disease of the circulatory system.

ICD 10 codes I613, I619, I629, I634 and I639 defined stroke.

### Predictors and study outcomes

In this paper, we studied the outcomes ACM, a composite of either death from cancer or incidence of cancer, and a composite of either death from CVD or incidence of CVD.

CVD included coronary heart disease (angina pectoris and myocardial infarction), cerebrovascular (cerebral infarction, cerebral haemorrhage, transitory ischaemic attack), claudicatio intermittens and CV death.

We studied the association of these outcomes with country of birth, adjusting for age, sex, anthropometrical measures (BMI) and lifestyle (smoking, intake of fruit and berries and/ or vegetables and alcohol consumption). When studying incidence of CVD morbidity and mortality we excluded individuals reporting a history of CVD and those with a diagnosis of old myocardial ischemia (ICD 10 code of I252) registered after the date of inclusion. Furthermore, we also excluded individuals reporting a history of CVD and those diagnosed with late effects of cerebral infarction (ICD 10 code I693).

When studying the incidence of cancer events and cancer death, we excluded individuals with a prior history of cancer diagnosis, or a cancer diagnosis registered prior to the date of study participation.

### Statistical analysis

Analyses were performed using IBM SPSS Statistics 28. Data are presented as means (standard deviation, SD), numbers (percentages) or for skewed data, medians (interquartile range, IQR). All tests were two-sided and a p-value of < 0.05 was considered statistically significant. Skewed variables were log-transformed before analysis to approximate normal distributions. The follow-up time was as described from the date of inclusion 2010–2012 until 31st of December 2018. Log-rank test was used to compare incidence rates and Cox regression for survival analysis. The continuous variables age and BMI were adjusted for assessing both linear and quadratic terms. The proportional hazard assumption was assessed in all the Cox regression models by adding an interaction term between follow up time and country of origin.

### Ethical considerations

All participants provided written informed consent and the Ethics Committee at Lund University approved the study (No. 2009/36, 2010/561 & 2019/01166). This investigation conforms to the principles outlined in the Declaration of Helsinki^30^.

## Results

The baseline characteristics of the Iraqi and the Swedish group are presented in Table 1. In Iraqis as compared to Swedes there was a male dominance and participants were younger (mean of 46.2 vs 49.6 years). The Iraqis had higher BMI levels, (29.3 vs 27.3), a higher prevalence of T2D, (12.8% vs 7.2%) and a higher prevalence of family history of T2D, (51.7% vs 27.8%). The Iraqis were also less insulin-sensitive and had lower levels of insulin secretion expressed as the medians of ISI and DIo, as well as were less physically active compared to the Swedish group. Only 246 (18.4%) Iraq born reported alcohol consumption of which 80% of alcohol consumers drank < 4 standard glasses per week. 626 (83.8%) of the Swedish born reported alcohol consumption, of which 71% of alcohol consumers drank ≤ 4 standard glasses per week.

**Table 1:** Characteristics of study participants (Iraqi-and Swedish-born living in Malmö).

Variable	Country of birth		Entire cohort	p-value
	Iraq (1398)	Sweden (757)	2155	
Age (years)	46.2 (11.1)	49.6 (9.5)	47.4 (10.3)	< 0.001
Male sex, n (%)	778 (58.3)	395 (52.9)	1173 (56.3)	0.017
BMI (kg/m^2^)	29.3 (4.5)	27.3 (4.7)	28.6 (4.7)	< 0.001
Smoking^1^, n (%)	320 (24.4)	193 (26)	513 (25)	0.410
Blood pressure lowering agents, n (%)	164 (12.6)	114 (15.4)	278 (13.6)	0.074
Hypertension^2^	165 (12.5)	118 (16)	283 (13.8)	0.026
Systolic blood pressure (mmHg)	133 (16)	135 (15)	134 (16)	0.27
Diastolic blood (mmHg)	80 (11)	82 (10)	81 (11)	0.12
T2D^3^, n (%)	171 (12.8)	54 (7.2)	225 (10.8)	< 0.001
T2D duration in years (SD)	2.0 (2.1)	1.98 (2.6)	2.0 (2.2)	0.9
T2D treatment, insulin (%)	7 (13)	18 (11.1)	25 (11.6)	0.8
T2D treatment, GLM^4^ (%)	89 (54.9)	16 (29.6)	105 (48.6)	0.002
Cancer^5^, n (%)	23 (1.7)	29 (3.9)	52 (2.5)	0.002
CVD^6^, n (%)	51 (3.8)	48 (6.4)	99 (4.8)	0.007
Total cholesterol (mmol/L)	4.9 (1.0)	5.2 (1.0)	5.0 (1.0)	0.382
p-LDL (mmol/L)	3.2 (0.8)	3.3 (0.9)	3.2 (0.9)	0.340
p-HDL (mmol/L)	1.2 (0.3)	1.4 (0.5)	1.3 (0.4)	0.004
p-Triglyceride (mmol/L)	1.6 (1.0)	1.3 (0.8)	1.5 (0.9)	0.680
Use of lipid lowering agents, n (%)	85 (6.5)	64 (8.6)	149 (7.3)	0.076
ISI (mmol/L*mIE/L−1)^7^	77.0	102.2	84.9	< 0.001
DIo (mmol/L*mmol/L*mmol/L)^7^	12,706.0	14,655.1	13,283.1	0.006
Waist circumference, cm	96.7 (11.2)	93.8 (13.5)	95.6 (12.2)	< 0.001
Physical activity^8^	1.9 (2.1)	4.0 (2.5)	2.6 (2.4)	< 0.001
Family history of diabetes, n (%)	690 (51.7)	208 (27.8)	898 (43.6)	< 0.001
Diet^9^, n (%)	105 (7.9)	91 (12.2)	196 (9.5)	0.002
Alcohol, n (%)	239 (17.9)	606 (81.1)	845 (40.6)	< 0.001

Crude data are presented as means (SD) or as numbers (percentages); family history refers to parents, children and/or siblings; LDL/HDL is low-density/high-density lipoprotein.

^1^Current tobacco smoking, ^2^hypertension at baseline, ^3^T2D at baseline, ^4^glucose lowering medication, ^5^cancer disease at baseline, ^6^CVD at baseline, ^7^differences in medians between groups, ^8^hours physically active per week, ^9^daily intake of fruit, berries and/or vegetables.

The ACM risk was significantly lower in the Iraqi group compared with the Swedish group (0.7% vs. 3.3%, p < 0.001). Moreover, cancer risk was also lower in the Iraqi group (2.1% vs. 8.1%, p < 0.001) when compared with the Swedish group. There were no significant differences in CVD risk and CSM in cancer and CVD in Iraqi compared with Swedish born.

**Figure 2:**
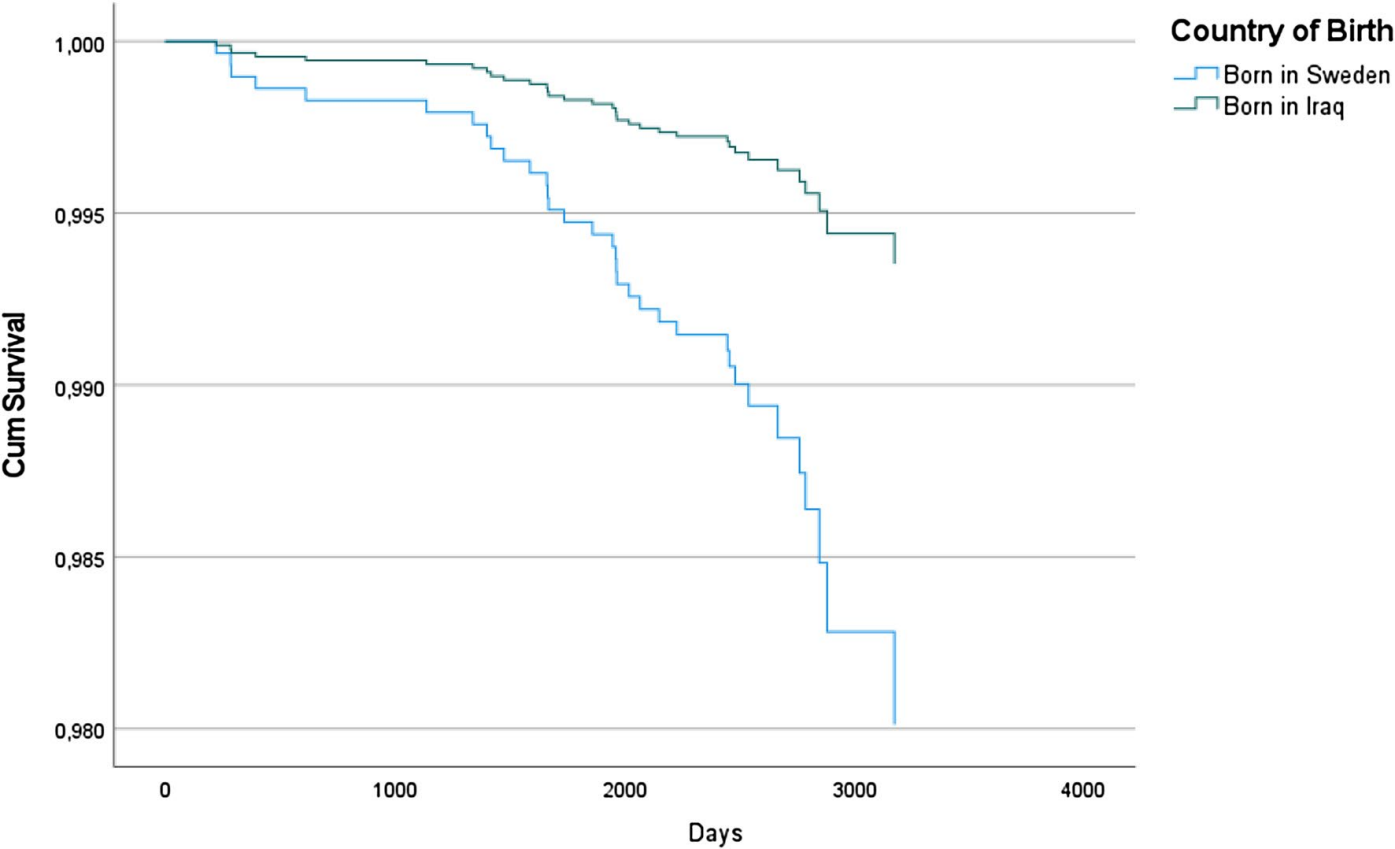
Time to event defined as ACM in a fully adjusted model.

In a Cox regression analysis, the HR of ACM was 68% lower in Iraqi immigrants than in native-born Swedes (HR = 0.32; 95% CI 0.13-0.79, p < 0.05) after adjustments for country of origin, age, sex, BMI, the diagnosis of T2D, CVD, cancer at baseline and lifestyle behaviours (Table 2). Figure 2 shows the survival rate in the fully adjusted model of Table 2 (model #5).

**Table 2:** Cox regression models showing adjusted HR of ACM, Model 1–5, 95.0% CI for Exp(B).

	Model 1	Model 2	Model 3	Model 4	Model 5
Born in Iraq	0.26 (0.12–0.54)***	0.41 (0.19–0.87)**	0.38 (0.18–0.83)**	0.38 (0.17–0.83)*	0.32 (0.13–0.79)*
Age^1^ (years)		0.98 (0.71–1.35)	0.96 (.69–1.32)	0.93 (0.68–1.29)	0.89 (0.63–1.22)
		1.00 (0.99–1.00)	1.00 (1.00–1.04)	1.00 (1.00–1.04)	1.00 (1.00–1.01)
Sex (male)		1.34 (0.67–2.68)	1.44 (0.71–2.93)	1.41 (0.70–2.87)	1.31 (0.63–2.72)
BMI^2^			1.03 (0.65–1.64)	0.99 (0.62–1.59)	1.04 (0.64–1.68)
			1.00 (0.99–1.01)	1.00 (0.99–1.01)	1.00 (0.99–1.01)
T2D^3^				1.41 (0.61–3.28)	1.43 (0.62–3.31)
CVD^4^				2.17 (0.92–5.10)	1.94 (0.83–4.53)
Cancer^5^				1.69 (0.62–4.63)	1.80 (0.65–4.94)
Smoking^6^					2.38 (1.16–4.90)*
Diet^7^					1.71 (0.85–4.70)
Alcohol					0.65 (0.30–1.35)

^1^Linear age and age squared, ^2^linear BMI and BMI squared, ^3^T2D at baseline, ^4^CVD at baseline, ^5^cancer disease at baseline, ^6^current tobacco smoking, ^7^daily intake of fruit, berries and/or vegetables, p < 0.05*, p < 0.01**, p < 0.001***.

**Figure 3:**
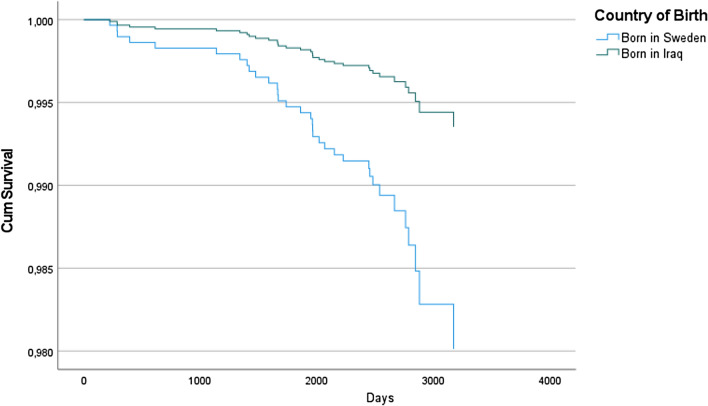
Time to event defined as cancer and death from cancer in a fully adjusted model.

Further we found a lower incidence of cancer morbidity and mortality in Iraqi-born as compared to Swedish-born individuals. The HR was 61% lower (0.39; CI 0.22–0.69, p < 0.01) in the fully adjusted model 5 (Table 3, Fig. 3). In Fig. 2 the event is defined as cancer or death from cancer.

**Table 3:** Cox regression models showing adjusted HR of cancer events and death from cancer, Model 1–5, 95.0% CI for Exp(B).

	Model 1	Model 2	Model 3	Model 4	Model 5
Born in Iraq	0.28 (0.18–0.44)***	0.37 (0.23–0.59)***	0.34 (0.21–0.55)***	0.35 (0.22–0.57)***	0.39 (0.22–0.69)**
Age^1^ (years)		1.31 (1.05–1.00)**	1.32 (1.05–1.65)**	1.32 (1.05–1.65)*	1.31 (1.04–1.64)*
		1.00 (0.99–1.00)	1.00 (0.99–1.00)	1.00 (0.99–1.00)	1.00 (0.99–1.00)
Sex (male)		0.85 (0.56–1.31)	0.81 (0.53–1.25)	0.80 (0.52–1.23)	0.74 (0.47–1.17)
BMI^2^			1.25 (0.81–1.92)	1.23 (0.80–1.90)	1.39 (0.85–2.26)
			1.00 (0.99–1.00)	1.00 (0.99–1.00)	1.00 (0.99–1.00)
T2D^3^				0.72 (0.36–1.44)	0.77 (0.38–1.53)
CVD^4^				1.78 (0.90–3.50)	1.77 (0.89–3.52)
Smoking^5^					1.10 (0.67–1.81)
Diet^6^					1.31 (0.84–2.05)
Alcohol					1.05 (0.62–1.80)

^1^Linear age and age squared, ^2^linear BMI and BMI squared, ^3^T2D at baseline, ^4^CVD at baseline, ^5^current tobacco smoking, ^6^daily intake of fruit, berries and/or vegetables, p < 0.05*, p < 0.01**, p < 0.001***.

**Figure 4:**
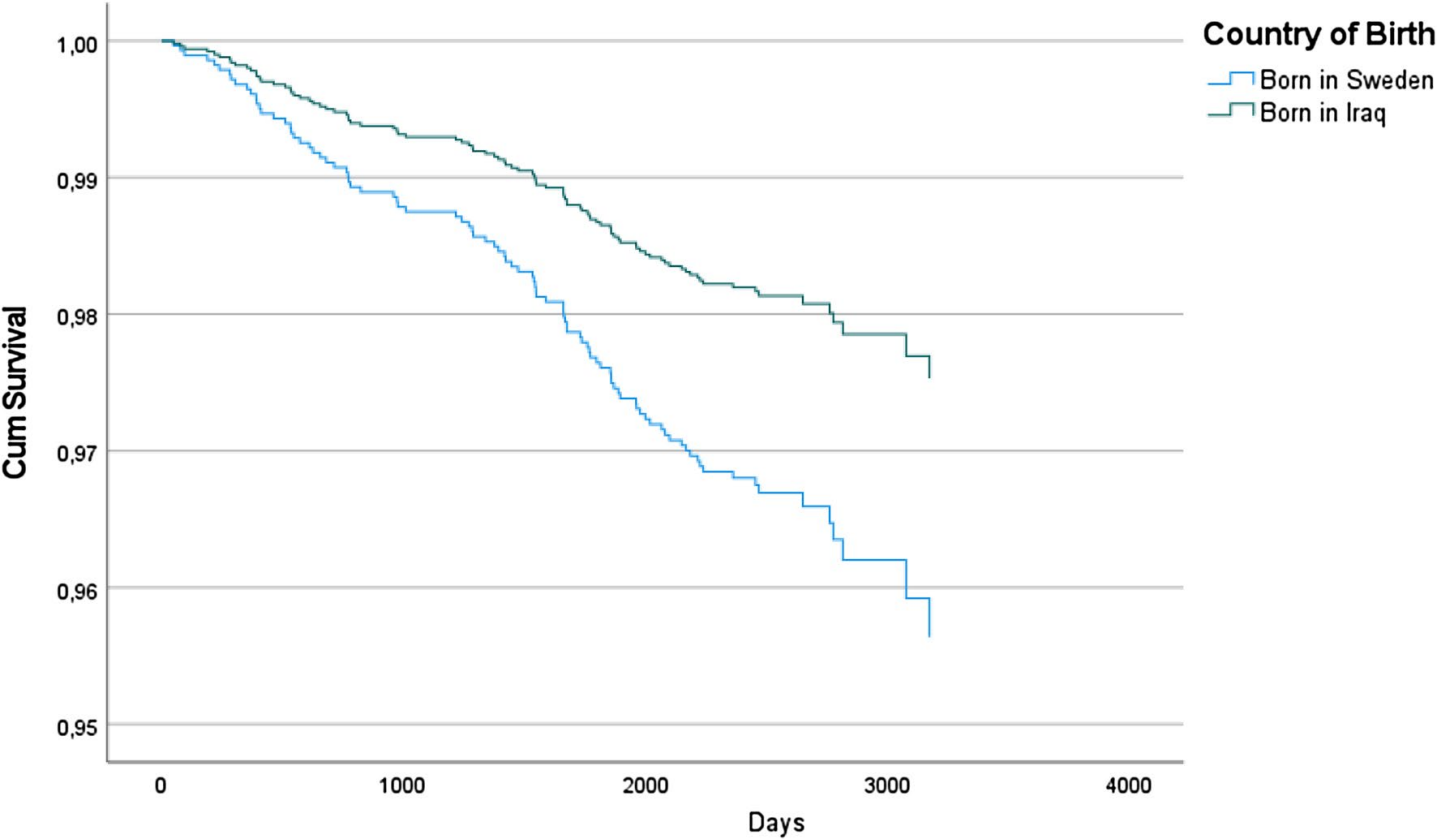
Time to event defined as CVD and death from CVD in a fully adjusted model.

A 44% lower incidence of CVD morbidity and mortality in Iraqi-born as compared to Swedish-born individuals was observed (HR 0.56; CI 0.33–0.95, p < 0.05) in the fully adjusted model #5 (Table 4, Fig. 4). The event in Fig. 4 is defined as CVD (coronary heart disease (angina pectoris and myocardial infarction), cerebrovascular (cerebral infarction, cerebral haemorrhage, transitory ischaemic attack), claudicatio intermittens or CV death.

**Table 4:** Cox regression models showing adjusted HR of CVD events or death from CVD, model 1–5, 95.0% CI for Exp(B).

	Model 1	Model 2	Model 3	Model 4	Model 5
Born in Iraq	0.61 (0.41–0.90)*	0.82 (0.54–1.22)	0.74 (0.49–1.12)	0.66 (0.43–1.02)	0.56 (0.33–0.95)*
Age^1^ (years)		1.49 (1.18–1.88)***	1.47 (1.17–1.86)**	1.45 (1.15–1.84)**	1.43 (1.13–1.82)**
		1.00 (0.99–1.00)**	1.00 (0.99–1.00)*	1.00 (0.99–1.00)*	1.00 (0.99–1.00)*
Sex (male)		1.53 (1.00–2.32)*	1.50 (0.98–2.28)	1.48 (0.97–2.27)	1.55 (0.99–2.43)
BMI^2^			1.44 (0.94–2.22)	1.49 (0.96–2.32)	1.53 (0.97–2.41)
			0.99 (0.99–1.00)	0.99 (0.99–1.00)	0.99 (0.99–1.00)
T2D^3^				1.80 (1.10–2.97)*	1.72 (1.04–2.86)*
Hypertension^4^				1.09 (0.67–1.77)	1.21 (0.74–1.98)
Cancer^5^				1.37 (0.58–3.21)	1.42 (0.61–3.34)
Smoking^6^					1.50 (0.97–2.33)
Diet^7^					1.07 (0.71–1.62)
Alcohol					0.75 (0.45–1.24)

^1^Linear age and age squared, ^2^linear BMI and BMI squared, ^3^T2D at baseline, ^4^hypertension at baseline, ^5^cancer at baseline, ^6^current tobacco smoking, ^7^daily intake of fruit, berries and/or vegetables, p < 0.05*, p < 0.01**, p < 0.001***.

## Discussion

Our data shows that despite the heavy burden of cardiometabolic, this 8-year follow-up study found that ACM, CVD and cancer morbidity, and CSM rates (in CVD and cancer) were lower in Iraqi-born immigrants compared to native-born Swedes.

Our findings are consistent with earlier data showing increased survival rate and lower cancer morbidity and mortality among immigrants compared to native populations^31–33^. In a Swedish observational cohort study of 62 557 individuals with hypertension with and without diabetes diagnosed 2001–2008, immigrants (with the exception of Finnish born) were shown to have lower mortality rate^13^. Our data are also consistent with nationwide data of people with new onset T2D showing survival advantage among first generation immigrants with T2D compared to Swedish-born. The ACM advantage among immigrants converged toward the host population in second generation immigrants, which is possibly due to acculturation to a western lifestyle^14^. Survival advantages in immigrants have been further observed in a Danish register-based study where refugees and reunification migrants had better survival from CVD compared to Danish-born individuals^34^. However, the strength of our study compared to the above is that our study also accounts for BMI, lifestyle behaviours and other well-known cardiometabolic risk factors that are not possible to adjust for in register-based studies.

The lower ACM rates amongst non-western immigrants is rather an epidemiological paradox considering the high burden of insulin resistance, obesity, sedentary lifestyles, T2D and socioeconomic vulnerability which, altogether, contribute to high cardiometabolic morbidity and mortality. The term ‘healthy migrant effect’ was therefore coined to address the fact that recent migrants seem to be in better health than natives from the host country^35^. The healthy migrant effect can potentially be countered by the so-called salmon bias explaining that people might choose to return to their country of origin when in a palliative state thus affecting the epidemiological data towards a lower mortality rate among immigrants in their host country and remain in the public registers in their adopted country, which can skew the figures^36,37^. However, this might not be the case for some immigrant groups including immigrants from Iraq, who have migrated from war-torn areas with unstable political climates, where the healthcare availability and quality of clinical care provided is more limited^38^. Aside from that, the Iraqi immigrants do not really fulfil the definition of “healthy” as they have a prevalence of T2D double that of Swedish-born as well as a higher prevalence of obesity, insulin resistance and higher prevalence of poor self-rated health as compared to Swedish-born^5,39^. Hence the healthy immigrant paradox is far from being fully understood and more research is needed to study eventual mental health benefits among immigrants and eventual correlation to the lower ACM rates observed among immigrants^40^. The fact that the mortality rates of second-generation immigrants merge toward the host population suggests possible gene-environment interactions may occur during adaptation to western lifestyles^14^.

There are only a few studies that address the incidence of cancer among immigrants from the Middle East region compared to that of the endogenous population. In the US, Arabic females tend to have a higher risk of thyroid cancer than Hispanic, Black Americans and non-Hispanic Whites; Arab Americans also have higher rates of liver, stomach and bladder cancer while Arabic males tend to have lower incidence rates of lung and prostate cancer than Black Americans^15,16^. As for immigrants to Sweden, a register study of the Swedish Family-Cancer Database (1958–1998) addressing cancer risk in first-generation immigrants has shown that cancer incidence varies greatly, with the lowest cancer incidence found among Iraqi men and Arab women^17^. Only 2–10% of cancers can be linked to a mutation in a particular gene, thus most cancer-causing factors may be linked to individual behaviour or are environment-related^33^. Further studies are needed to identify eventual genetic risk factors as well as the cancer incidence among second- generation immigrants and whether the healthy migrant effect decreases depending on the number of years spent living in Sweden.

Studies on CVD morbidity and mortality among migrant populations compared to host populations have shown that—the longer duration of residence in the host countries – migrants have similar or higher rates of CVD compared to those of the host populations^25^. A systematic review of publications between 2000 and 2014 addressing the risk of CVD among immigrants to high-income countries showed that Middle Eastern migrants in Western Europe had similar or higher rates of CVD than the host populations^41^. Our findings of lower rates of CVD among Iraqi immigrants compared to the native Swedish control group could be explained by a shorter follow-up time. Studies with longer follow up time are needed to study how the incidence rates of CVD change over time with a longer duration of stay in the host country.

Fruit and vegetable intake has been shown to reduce the risk of cancer, ACM and CVD in a systematic review and a meta-analysis^42^. In this paper adjustments have been made to daily intake of fruit, berries and/ or vegetables when studying differences between the groups in ACM, cancer morbidity and mortality and CVD morbidity and mortality. The association between alcohol consumption and CVD is rather complex and the associations are modulated by the amount and pattern of alcohol consumed, low-to-moderate daily alcohol consumption (1–2 standard drinks) has been associated with a reduced risk of CVD and ACM^43^. In this paper a binary variable of alcohol consumption has been chosen due to most of the participants reporting alcohol consumption, consume ≤ 4 standard glasses per week also only 18.4% of the Iraq born participants reporting alcohol consumption.

### Strengths and weaknesses of the study

Much of the epidemiological research providing information on cardiometabolic incidence rates among immigrants is registry-based and provides estimates without more specific adjustments beyond age and sex. The MEDIM cohort is unique in that it is thoroughly phenotyped consisting of data being collected through health examinations, questionnaires, fasting samples and OGTT conducted by trained research nurses. This has enabled us to study the impact of ethnicity on ACM, cancer morbidity and mortality with adjustment for anthropometrical measures, glucose regulation and lifestyle. The participants were randomly selected from the Swedish Population Register. As reported previously the study sample was representative compared to the background population^44^. The accessibility to health care is equal for both immigrants and native Swedes, which is another strength of this study. Thus, access to health care could not explain the differences observed in the incidence rates of cancer. A potential limitation of the study is the uneven sex and age distribution with the Iraqis being younger and Iraqi men participating to a higher degree in the study. This might have underestimated the rate of breast and gynaecological cancers. Another limitation is the short follow-up time, and the low number of cause specific deaths that contributes to imprecise estimates, particularly given the less reliable nature of information from death certificates regarding actual cause of death. Another limitation is that we have not studied different types of cancer diagnosis, which is due to the limited number of cases for each cancer type. In this study, we focused on one immigrant group, and the study findings might not be generalisable to other non-western migrant groups in Sweden.

### Representativeness of the study sample

The age and sex distribution in participating immigrants from Iraq did not differ compared to the eligible background population. The Swedish-born participants were older (49.3 vs. 45.5 years) but the sex distribution did not differ from the eligible native Swedish population. The prevalence of T2D did not differ compared to the eligible background population thus indicating a representative study sample^5^.

### Unanswered questions and future research

More studies are needed that address possibly environmental, genetic, or hormonal factors putting the native Swedish group at higher risk of cancer morbidity and mortality. Longitudinal studies are also needed to study whether the risk of ACM remains at lower rates in non-western immigrants or merge towards the rates of the Swedish-born host population with longer duration of stay.

## Conclusion

We conclude that Iraqi-born immigrants have lower ACM rates than Swedish-born as well as lower rates of cancer and CVD morbidity and mortality. The differences in mortality and cancer rates across European and Middle Eastern ethnicities are not fully explained by age, sex, anthropometrical measures, glucose regulation and lifestyle but lie elsewhere. Further studies are needed to increase the understanding of contributing mechanisms. Studying ethnical differences in risk or protective factors in migrant groups is of great importance considering the increasing migrant populations in western countries.

## Data Availability

The datasets used and/or analysed during the current study available from the corresponding author on reasonable request.

## Abbreviations

ACM: All-cause mortality
BMI: Body mass index
CV: Cardiovascular
CVD: Cardiovascular disease
CSM: Cause specific mortality
DIo: Oral disposition index
HDL: High-density lipoprotein
ICD: International Classification of Disease
IQR: Interquartile range
ISI: Insulin sensitivity index
LDL: Low-density lipoprotein
SD: Standard deviation
T2D: Type 2 diabetes
